# Synthesis of Gold Clusters and Nanoparticles Using Cinnamon Extract—A Mechanism and Kinetics Study

**DOI:** 10.3390/molecules29071426

**Published:** 2024-03-22

**Authors:** Magdalena Luty-Błocho, Jowita Cyndrowska, Bogdan Rutkowski, Volker Hessel

**Affiliations:** 1 AGH University of Krakow, Faculty of Non-Ferrous Metals, al. A. Mickiewicza 30, 30-059 Krakow, Poland; 2 AGH University of Krakow, Faculty of Metals Engineering and Industrial Computer Science, al. A. Mickiewicza 30, 30-059 Krakow, Poland; rutkowsk@agh.edu.pl; 3 School of Chemical Engineering, The University of Adelaide, Adelaide, SA 5005, Australia; volker.hessel@adelaide.edu.au

**Keywords:** chemical reduction, nucleation and autocatalytic growth, Watzky—Finke model, ultra-small particles, gold clusters, cinnamon extract, dynamic equilibrium

## Abstract

In this work, UV-Vis spectrophotometry, High Resolution Scanning Transmission Electron Microscopes and selected experimental conditions were used to screen the colloidal system. The obtained results complement the established knowledge regarding the mechanism of nanoparticle formation. The process of gold nanoparticles formation involves a two-step reduction of Au ions to Au(0); atom association and metastable cluster formation; autocatalytic cluster growth; ultra-small particle formation (1–2 nm, in diameter); particle growth and larger particles formation; and further autocatalytic crystal growth (D > 100 nm). As a reductant of Au(III) ions, a cinnamon extract was used. It was confirmed that eugenol as one of the cinnamon extract compounds is responsible for fast Au(III) ion reduction, whereas cinnamaldehyde acts as a gold-particle stabilizer. Spectrophotometry studies were carried out to track kinetic traces of gold nanoparticle (D > 2 nm) formation in the colloidal solution. Using the Watzky—Finke model, the rate constants of nucleation and autocatalytic growth were determined. Moreover, the values of energy, enthalpy and entropy of activation for stages related to the process of nanoparticle formation (Index 1 relates to nucleation, and Index 2 relates to the growth) were determined and found to be E_1_ = 70.6 kJ, E_2_ = 19.6 kJ, ΔH_1_ = 67.9 kJ/mol, ΔH_2_ = 17 kJ/mol, ΔS_1_ = −76.2 J/(K·mol), ΔS_2_ = −204.2 J/(K·mol), respectively. In this work the limitation of each technique (spectrophotometry vs. HRSTEM) as a complex tool to understand the dynamic of the colloidal system was discussed.

## 1. Introduction

Noble metals exhibit unique physicochemical properties [1,2] that depend on the metal (e.g., Ag, Au, Pd, Pt, Rh), particle morphology (shape, size) [3,4,5], surface properties (e.g., adsorption capacity of active compound) [6,7] and stability [8]. One of intriguing aspects of the process of noble metal nanoparticle formation is the ability to control their morphology. In the case of particles with a radius (R) above 2 nm [9], it can be realized by using proper process conditions, including the choice of the reducing agent, the reagent concentration, pH, temperature and a stabilizer. The proper selection of a reducing agent is an important point, especially in the process of synthesizing small and monodispersed particles. In this case, the choice of a reductant is strongly limited to sodium borohydride [10]. The application of NaBH_4_ leads to “burst” nucleation [11], protecting against further particle growth. However, sodium borohydride is dangerous and toxic [12]; therefore, the application of other efficient and “green” reductants is desirable from the point of view of medical applications or syntheses using harmless reagents.

Recently, a new trend related to the application of metal clusters has appeared (see details coming from Scopus analysis, paragraph 1, Supplementary Materials). The growing interest in gold nanoclusters is related to unique molecule-like properties and the biocompatibility of AuNCs. An AuNC is a small object which consists of several to 100 gold atoms and a diameter below 2 nm [9]. In contrast to particles of a larger size (>2 nm), the AuNCs have no plasmon resonance effect. Moreover, AuNCs can be considered as molecules due to the effects of quantum confinement, in which the conduction band is significantly quantized. Gold clusters containing a core size comparable to the Fermi wavelength of an electron, i.e., 5 Å, are characterized by fluorescence. This effect is observed as a result of absorbing light of a certain energy. Consequently, electrons are excited and move to higher energy levels, releasing energy in the form of light, albeit with a lower energy than the length of light that causes excitation. Considering that AuNCs absorb light in a wavelength range from 650 to 900 nm (known as a therapeutic window of tissues), they find an application in, for example, cancer diagnostics [9]. Most of the articles (see Figure S1, Supplementary Materials) focus on gold nanoclusters application in the context of fluorescence properties rather than kinetics and the mechanism of their formation. However, this basic knowledge can be helpful in better understanding the process of nanoparticle formation, including the formation of clusters, as well as ultra-small and larger particles.

A common model describing the mechanism of nanoclusters formation has been suggested by Watkzy and Finke (W—F model). The W—F model was proposed in 1997 [13] for the formation of iridium nanoclusters by reducing its salt with hydrogen. The process of iridium clusters formation consisted of slow continuous nucleation and fast autocatalytic growth. These two stages were described as two reactions:

(1) $$nIr(0)\overset{k_{1}}{\to }Ir{\left(0\right)}_{n}$$

(2) $$Ir(0)+Ir{\left(0\right)}_{n}\overset{k_{2}}{\to }Ir{\left(0\right)}_{n+1}$$

These reactions have been simplified to:

(3) $$A\overset{k_{1}}{\to }B$$

(4) $$A+B\overset{k_{2}}{\to }2B$$

where: *A*—cluster precursor, i.e., *nIr*(0)—“*n*” *Ir* atoms, *B*—active cluster surface, i.e., *Ir*(0)*_n_* in Equation (1), *Ir*(0)*_n_*_+1_—product of reaction (2), *k*_1_—rate constant of nucleation, *k*_2_—rate constant of autocatalytic growth.

Equations (3) and (4) are more universal and can be used to describe any two-stage process in which the first one is slow and the second one is autocatalytic.

The W—F model was the first model, which connects mathematical and physical features of observed processes in the form of a sigmoidal kinetic curve (a dependence of the concentration in time). Since that time, this model was also successfully applied for noble metals (Au, Ag, Pd, Pt, etc.), nanoparticle nucleation and growth description [14].

In some cases, for the description of gold nanoparticles formation using hydrate hydrazine, ascorbic acid and glucose, the W—F model was expanded by the addition of one more stage related to metal reduction, i.e., Au(III) to Au(I) [15,16,17]. The following steps were analogous to the original W—F model (Equations (3) and (4)). Hornstein et al. [18] added bimolecular aggregation as a third step to the W—F model:

(5) $$P+P\overset{k_{3}}{\to }P_{L}$$

where: *P*—small particles; *P_L_*—large particles, *k*_3_—rate constant of bimolecular aggregation.

Besson et al. [19] proposed a fourth step in which autocatalytic agglomeration between small (*P*) and larger, bulk-metal-like (*P_L_*) particles was considered. In this work, the W—F model in its basic form, Equations (3) and (4) was used. To rule out the problem of separating the nucleation and growth steps over time in the graph obtained after the sigmoidal curve linearization process, the Bentea et al. [20] approach to facilitating the determination of kinetic rate constants using the tools available in the Origin Pro 8.5 software was applied.

Moreover, we show unexpected results related to the synthesis of nanoparticles by chemical reduction with cinnamon extract, which allowed us to observe metastable clusters, cluster aggregates, as well as ultra-small and 10–20 nm particles (sample with the same composition, but different synthesis temperature). These results give more insight into the process of cluster and particle formation. Gold nanoparticles were synthesized via a chemical reduction method in which Au(III) ions were reduced and stabilized using cinnamon extract [21]. This synthesis might be a promising alternative method for the production of ultra-small gold (1–2 nm in diameter) particles for catalytic and medical applications. Moreover, the role of eugenol and cinnamaldehyde as the main components of cinnamon extract in the process of gold nanoparticles formation was considered.

## 2. Results and Discussion

### 2.1. Experimental Conditions

The process of slow nucleation and fast autocatalytic growth was carried out in the temperature range from 20 to 60 °C. The cinnamon extract was freshly prepared prior to the synthesis.

### 2.2. Preview of the Course of the Reaction

Spectrophotometry is a relevant technique that allows for the following reaction course, both at the stage of reduction of metal ions, as well as the intermediate and final products. Moreover, it can help to identify the reacting components. In the case of an aqueous solution of Au(III), hydrolysis progress [22], intermediates (the appearance of Au(I) ions), complex formations [23], etc. can be detected. However, in the case of cinnamon extract, it is possible to qualitatively and/or quantitatively distinguish its components [24].

**Figure 1:**
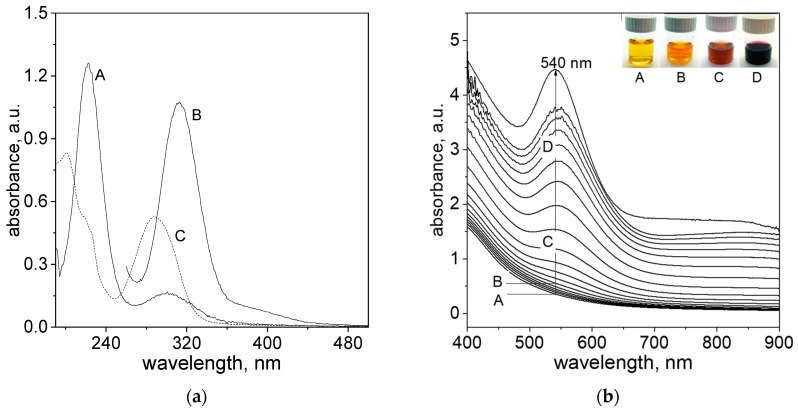
Spectra of reagents, i.e., an aqueous solution of Au(III) ions and cinnamon extract (Cex). Conditions: C_0, Au(III)_ = 0.2 mM (A); C_0, Au(III)_ = 1.0 mM (B); C_0,Cex_ = 20 g/L (diluted 100 times in deionized water, C), T = 20 °C (**a**); Spectra evolution after reagents mixing, i.e., Au(III) ions and cinnamon extract (**b**). Samples notation: A—1 min, B—5 min; C—10 min and D—15 min later. Conditions: C_0, Au(III)_ = 0.01 M, C_0,Cex_ = 18 g/L, T = 20 °C (**b**).

Thus, before gold nanoparticles synthesis, the spectra of reagents, i.e., an aqueous solution of metal precursor and cinnamon extract, were recorded (see Figure 1a).

**Figure 2:**
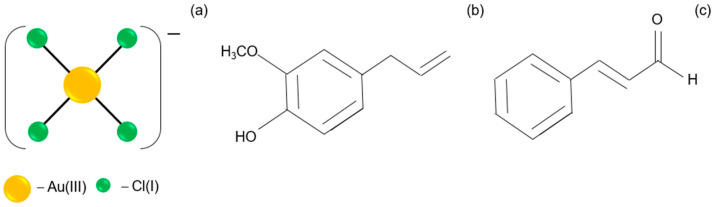
The structures of reagents: [AuCl_4_]^−^ (**a**); eugenol (**b**); cinnamaldehyde (**c**).

The solution of Au(III) ions (the molecular structure shown in Figure 2a) has a yellow color and exhibits a characteristic spectrum with two maxima at 220 and 302 nm (Figure 1a, A), indicating the progress of hydrolysis [22]. For higher Au(III) ion concentrations (sample B, Figure 1a), the spectrum has a strong peak at 314 nm, indicating the presence of an [AuCl_4_]^−^ complex (Figure 2a), whereas an aqueous solution of the cinnamon extract absorbs light below 400 nm. When diluting the cinnamon extract to concentration 0.2 g/L, the spectrum exhibits two characteristic maxima, with the first one registering at 200 nm and the second one at 282 nm (sample C, Figure 1a).

Extracts from natural products contain multiple molecular components. These components may interact with each other during synthesis in addition to the reaction product. This is different from the process that uses industrial chemicals with only one well-defined reagent, which might be of an advantage or disadvantage for the final nanoproduct. Gao et al. [25] report that the cinnamon extract contains several active ingredients, including eugenol (Figure 2b), cinnamaldehyde (Figure 2c), cinnamic acid and coumarin, depending on the part of the plant from which it was obtained.

Rao et al. [26] reported that cinnamon extract originating from a bark basically contains 65–80% cinnamaldehyde, giving a pale-yellow color to the solution, with 5–10% eugenol (colorless); therefore, we have verified which components might be assigned to an obtained spectrum of cinnamon extract. The peaks shown in Figure 1a can be assigned to the eugenol, which is one of the main compounds of the cinnamon extract. Moreover, the position of these maxima is in good agreement with the literature data [27,28]. However, the presence of cinnamaldehyde cannot be neglected.

Cox et al. [29] found maxima at 200, 225 and 290 nm assigned to cinnamaldehyde. To confirm the presence of this compound, we performed a spectrum deconvolution using the Gaussian model in Origin Pro 8.5 software. The obtained spectrum (Supplementary Materials, Figure S2) shows three maxima located at 196, 225 and 289 nm. These results suggest that, in our case, both components (i.e., eugenol and cinnamaldehyde) are present and should be taken into account as possible reducing agents of Au(III) ions.

Based on obtained results (Figure 1a), it can be concluded that the spectra coming from metal ions and reductants overlap, and the process of Au(III) ions reduction to Au(I) is impossible to follow using UV spectrophotometry. However, in the visible range of the wavelength, it is possible to track the process of gold nanoparticle formation, as it was demonstrated in Figure 1b. After reagent mixing, the change in the spectra, colors of the colloidal solution and size of the obtained particles were registered. It was shown that the yellow color coming from the cinnamon extract (sample A (t = 1 min), Figure 1b) changes with time to orange (sample B (t = 5 min), Figure 1b), dark orange (sample C (t = 10 min), Figure 1b) and, finally, to dark red (sample D (t = 15 min), Figure 1b).

The fast appearance of the first nuclei, i.e., 5 min after the reagents were mixed (Figure 1b), allows us to conclude that the process of Au(III) to Au(I) ions reduction is very fast comparing to the next step, i.e., reduction Au(I) to Au(0). It is worth noting that the solution containing Au(I) ions is colorless and has no characteristic spectrum. Thus, this process cannot be observed in the UV region (i.e., below 400 nm), whereas the product of reaction, i.e., gold clusters formed via analogy to iridium clusters, Equations (1) and (2) can be observed using, for example, HRSTEM (see, Section 2.5). The next stages relate to ultra-small gold particle formation (D ≤ 2 nm), and their further growth leading to particle formation (more details in Section 2.7) can also be registered using HRSTEM, whereas spectrophotometry might be applied for tracking the progress of a reaction, but after reaching a particle of critical size (D > 2 nm). Considering the fact that the formation of nanoparticles contains slow and rapid (autocatalytic) steps, the W—F model finds its application for kinetic curve description (see, Section 2.3).

### 2.3. Determination of Slow Continuous Nucleation and Growth Rate Constants

**Figure 3:**
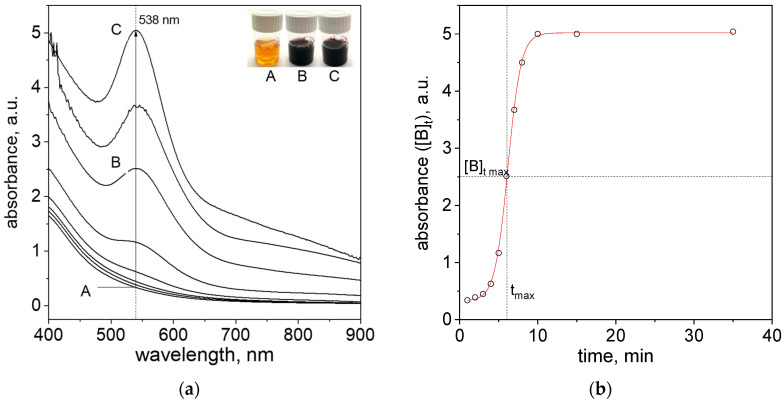
Spectra evolution and change of solution color (A—1 min later, B—6 min later, C—25 min later) after reagents mixing, i.e., Au(III) ions and cinnamon extract (**a**); Kinetic curve registered at 538 nm (**b**). Conditions: C_0, Au(III)_ = 0.01 M, C_0,Cex_ = 18 g/L, T = 40 °C.

After reagent mixing, a change in color in the solution (Figure 3a, Samples A–C) was observed. These changes were related to the progress of the nanoparticle formation process. During this process, the spectra evolution in visible wavelength range, with a visible LSPR peak located at the wavelength (λmax) = 538 nm, was recorded (Figure 3a).

From the obtained spectra (Figure 3a), the kinetic curve (absorbance vs. time) at 538 nm was drawn (Figure 3b). The characteristic of the obtained curve is sigmoidal and is typical for the process of noble metal nanoparticle formation [30]. This also confirms the correctness of the assumed model, Equations (3) and (4). The W—F model allows the determination of *k*_1_ and *k*_2_, which are the constants of the reaction rate. Knowing them, we can write the formula for the total reaction rate (6):

(6) $$-\frac{d[A]}{dt}=\frac{d[B]}{dt}=k_{1}[A]+k_{2}[A][B]$$

At the beginning of the reaction (*t* = 0) [*B*]_0_ = 0, so the sum of [*A*]*_t_* + [*B*]*_t_* = [*A*]_0_ for the entire duration of the process. Performing the differentiation of the kinetic Equation (6) we obtain the expression for [*A*]*_t_* and [*B*]*_t_*, which are given as:

(7) $${\left[A\right]}_{t}={\left[A\right]}_{0}\cdot \frac{k_{1}+k_{2}{\left[A\right]}_{0}}{k_{2}{\left[A\right]}_{0}+k_{1}e^{(k_{1}+k_{2}{\left[A\right]}_{0})t}}$$

(8) $${\left[B\right]}_{t}={\left[A\right]}_{0}-{\left[A\right]}_{t}$$

(9) $${\left[B\right]}_{t}={\left[A\right]}_{0}(1-\frac{k_{1}+k_{2}{\left[A\right]}_{0}}{k_{2}{\left[A\right]}_{0}+k_{1}e^{(k_{1}+k_{2}{\left[A\right]}_{0})t}})$$

In the process of precipitation of the metallic phase, the kinetic curve in the form of a sigmoid is recorded (function [*B*]*_t_* = f (t), see Figure 3b). The key condition is the assumption that *k*_1_ ≪ *k*_2_ [*A*]_0_, which allows for the separation of nucleation and growth processes. Bentea et al. [20] summarized the mathematical operation and equations which are needed for determination of rate constants of nucleation and particle growth determination from sigmoidal kinetic curves. Most important parameters, i.e., *t*_max_, ${\left[B\right]}_{t_{\max}}$, *t_in_*_(*jerk*)_ and corresponding formulas are summarized in Table 1.

In Figure 3b, the value of ${\left[B\right]}_{t_{\max}}$ was marked. The ${\left[B\right]}_{t_{\max}}$ relates to the absorbance value (according to the Lambert−Beer law, [*B*] ∝ concentration of particles in the solution) at *t*_max_. This time value can be determined by calculating the derivative of the function [*B*]*_t_* (Figure S4a, Supplementary Materials). The obtained graph exhibits the maximum corresponding to the time (*t*_max_) for which the process rate is the highest (Figure S4a, Supplementary Materials). The value of *t*_max_ corresponds to the value of ${\left[B\right]}_{t_{\max}}$ and is equal to ~2.5 (a.u.). These values are needed for the determination of observed rate constants and the value of [*A*]_0_ (Equations (10)–(12)), respectively. The determination of successive derivatives, including the third-order derivative, allows for the determination of the *t_in_*_(*jerk*)_ (this time representing a zero point in the jerk curve of the third derivative of *B* [20], Figure S4c, Supporting Materials) and thus needs a third equation allowing for the solution of Equations (10)–(12). The second derivative allows for *t_in_* (time of induction period) determination [20] (Figure S4b, Supplementary Materials).

The point of intersection with the x-axis at point 0 is the *t_in_*_(*jerk*)_ value and is 4.44 min at 40 °C (Figure S4c, Supporting Materials). The determined value of *t_in_*_(*jerk*)_ allows us to calculate the value of *k*_2_, which is 0.165 (a.u.)^−1^min^−1^. The value of the rate constant for the nucleation process (*k*_1_) was determined from the formula described by Equation (10) and equals 0.0056 min^−1^. The corresponding rate constants at other temperatures were determined in an analogous manner, and the calculated values of the rate constants for the nucleation process and the autocatalytic growth of gold nanoparticles, as well as *t*_max_ and *t_in_*_(*jerk*)_, are summarized in Table 2.

### 2.4. Thermodynamic Parameters Determination for Nucleation and Growth of Gold Nanoparticles

**Figure 4:**
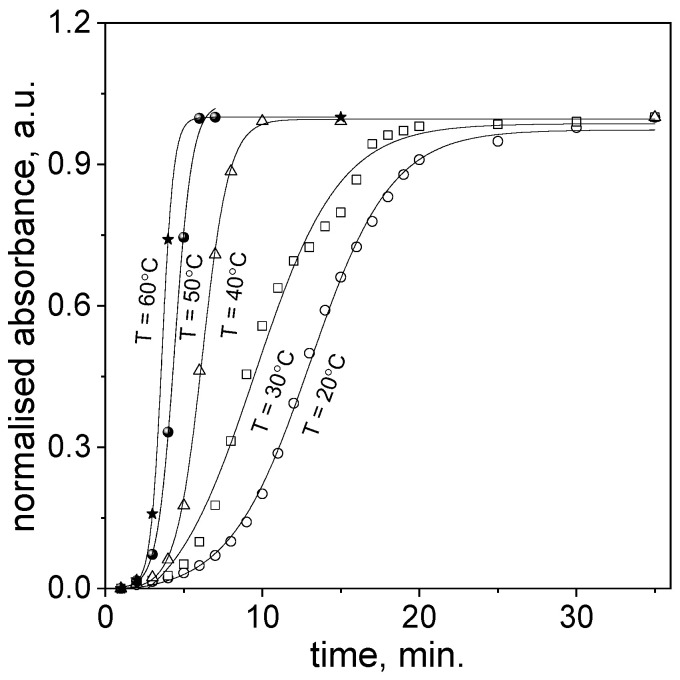
Kinetic curves for slow continuous nucleation and fast autocatalytic growth of gold nanoparticles obtained through Au(III) ion reduction using cinnamon extract. Conditions: C_0, Au(III)_ = 0.01 M, C_0,Cex_ = 18 g/L.

After reagents mixing, i.e., aqueous solutions containing Au(III) ions and cinnamon extract, at a temperature in the range 20–60 °C, the spectra in the visible range (450–600 nm) were recorded (samples shown in Figure 1b and Figure 3a). Based on these spectra the respective kinetic curves were determined (see Figure 4).

As can be expected, the process of nanoparticles formation runs faster at higher temperatures. The process of nucleation is shorter and the slope of sigmoidal curve increases. Based on the obtained kinetic curves at different temperatures, and using the approach described in Section 2.3, the values of *t*_max_, *t_in_*_(*jerk*)_ were determined from the first and third derivatives (examples shown in Figure S4a,c, Supporting Materials), and calculated values of *k*_1_ and *k*_2_ from Equations (10) and (12) (see, Table 2) were obtained.

The values of the rate constants (Table 3) were used for thermodynamic parameter determinations. The activation entropy (Δ*S*) and activation enthalpy (Δ*H*) for the nucleation and growth processes were determined from the linear form of the Eyring equation:

(13) $$\ln\frac{k}{T}=(\ln\frac{k_{B}}{h}+\frac{\Delta S}{R})-\frac{\Delta H}{RT}$$

where: *k*—the rate constants of nucleation (*k*_1_) and autocatalytic growth (*k*_2_), respectively, *T*—temperature in Kelvin; *R*—gas constant (8.314 J/(mol·K)); *k_B_*—Boltzmann constant; *h*—Planck’s constant, Δ*S*—activation entropy of nucleation (Δ*S*_1_) and autocatalytic growth (Δ*S*_2_), respectively; Δ*H*—activation enthalpy of nucleation (Δ*H*_1_) and autocatalytic growth (Δ*H*_2_), respectively.

Also, the values of activation energy of nucleation and growth were determined from a linear form of the Arrhenius equation:

(14) $$\ln k=(\ln A)-\frac{E_{a}}{RT}$$

where: *k*—the rate constants of nucleation (*k*_1_) and autocatalytic growth (*k*_2_), respectively, *T*—temperature in Kelvin; *R*—gas constant (8.314 J/(mol·K)); *A*—pre-exponential factor; *E_a_*—activation energy, J.

**Figure 5:**
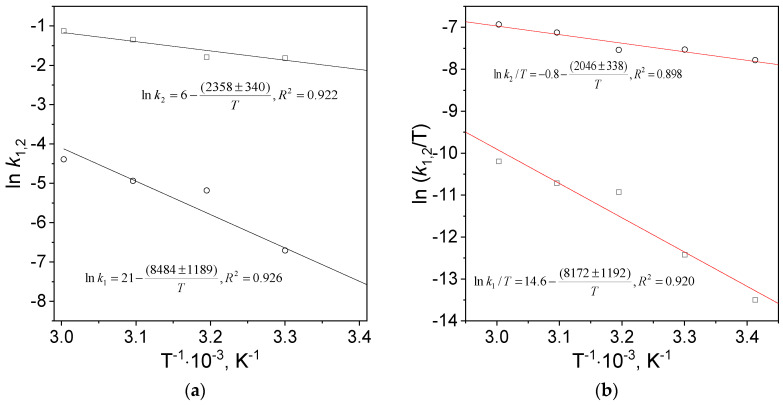
The Arrhenius (**a**) and Eyring (**b**) dependencies. Conditions: C_0, Au(III)_ = 0.01 M, C_0,Cex_ = 18 g/L, T = 20–60 °C.

The values of activation energy, entropy and enthalpy were determined from the slope and intercept of the linear equation (Figure 5) fitted to the experimental data, and they were gathered in Table 3.

Determined values of the activation parameters for nucleation and growth processes were compared in Table 3. The value of pre-exponential factor in Arrhenius equation for nucleation is seven orders of magnitude larger than that for the growth. The values of activation energy and enthalpy are similar for individual processes (nucleation and growth). The activation energy for nucleation is approximately four times higher than for the growth and is similar to the results obtained by Simeonova et al. [31]. Moreover, the calculated values of activation entropy for nucleation and growth are negative. In accordance with the literature [16,32,33] a negative value of activation entropy suggests association mechanisms, including nucleation and growth.

### 2.5. High Resolution Scanning Transmission Microscope for Gold Nanoparticles Characterization

**Figure 6:**
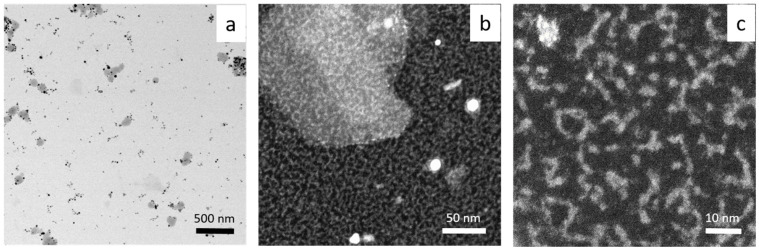
HRSTEM results registered at different magnification (**a**–**c**). Conditions: C_0, Au(III_) = 0.01 M, C_0,Cex_ = 18 g/L, T = 20 °C.

**Figure 7:**
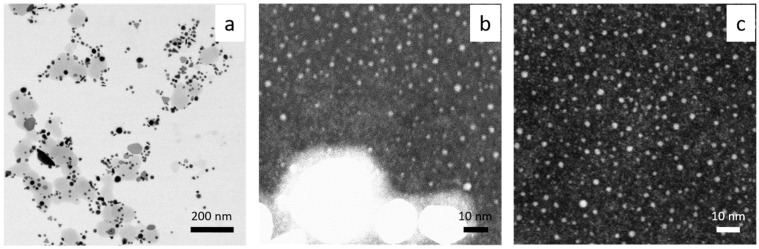
HRSTEM results registered at different magnification (**a**–**c**). Conditions: C_0, Au(III)_ = 0.01 M, C_0,Cex_ = 18 g/L, T = 60 °C.

The synthesized gold nanoparticles morphology (size, shape) was characterized using the HRSTEM technique (see Figure 6 and Figure 7).

HRSTEM shows that AuNPs synthesized at 20 °C are different in shape, with a diameter of 10–20 nm (Figure 6a,b). Additionally, they are very thin objects with elongated and irregular shapes (see Figure 6b,c). The same analysis was carried out for HRSTEM colloidal gold synthesized at a higher temperature (60 °C), see Figure 7.

Gold particles with irregular shapes (spherical, triangle, plate, rod) and diameters in the range 10–100 nm were found (Figure 7a). The use of higher magnification showed the presence of ultra-small particles with a diameter of 1–2 nm (Figure 7b,c), and a fluorescence study confirmed that this sample emits light. The maximum of the spectrum was registered at 455 nm (see, Supplementary Material, Figure S8), and the highest value of intensity was registered at a excitation wavelength of 320 nm. It is worth noting that such a spectrum was obtained only for diluted samples (10 times).

**Figure 8:**
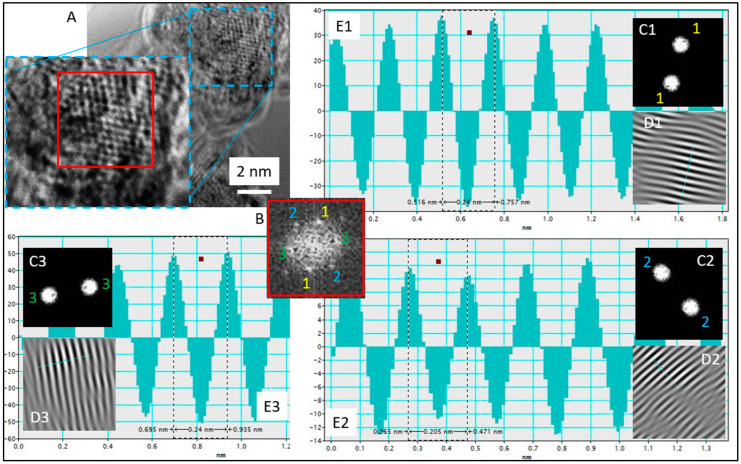
(**A**) HRTEM image with selected analyzed area, (**B**) FFT analysis with selected spots (1–1, 2–2, 3–3), (**C1**–**C3**) Mask used to select certain spots for IFFT, (**D1**–**D3**) IFFT image using indices chosen by (**C1**–**C3**) mask, (**E1**–**E3**) lattice parameter measurement through measuring the distance of white fringes on IFFT (**D1**–**D3**) images. Conditions: C_0, Au(III)_ = 0.01 M, C_0,Cex_ = 18 g/L, T = 60 °C.

Comparing results obtained at 20 and 60 °C, it can be concluded that using higher temperatures during the synthesis process leads to faster formation of gold nanoparticles. IFFT (Inverse Fast Fourier Transform) analysis of the lattice parameters confirmed the presence of gold. In both cases of the applied temperature, an interplanar distance of ~2.4 and ~2 Ǻ was found, which is strong evidence for the existence of Au planes of the {111} and {200} families, respectively (Figure 8, Supporting Materials Figure S9).

### 2.6. The Role of Eugenol and Cinnamaldehyde in the Process of Gold Nanoparticles Formation

Eugenol and cinnamaldehyde are considered as main components of cinnamon extract and can be responsible for Au(III) ion reduction. The role of each reducing agent on the process of AuNPs formation needs to be clarified. Srivastava et al. [34] consider eugenol as a dominant component of clove extract responsible for the reduction of Au(III) ions. The presented mechanism of reduction involves eugenol as a two electron reducing agent. Considering the protonation of eugenol [34], the reduction in Au(III) ions can be described by following scheme:

(15) $$Eug+Au\left(III\right)\overset{k_{R1}}{\to }Eug\left(ox\right)+Au(I)$$

(16) $$\frac{1}{2}Eug+Au\left(I\right)\overset{k_{R2}}{\to }\frac{1}{2}Eug\left(ox\right)+Au(0)$$

(17) $$Au(0)+Au\left(III\right)\overset{k_{R3}}{\to }2Au(0)$$

where: Eug—eugenol; Eug(ox)—oxidized form of eugenol; *k*_R1_—kinetic rates constant of Au(III) to Au(I) ions reduction; *k*_R2_—kinetic rates constant of Au(I) reduction to Au(0); *k*_R3_—kinetic rates constant of autocatalytic growth.

Srivastava et al. [34] showed that the process of forming AuNPs takes place within 20 min for a ratio of Au(III) to clove equal 1:1. With a lower amount of reductant, particle growth occurred within hours. The synthesized particles varied in shape (e.g., triangular shape) and size (50–300 nm) depending on the used ratio of clove to Au(III) ions (it is similar to our results shown in Figure 6a and Figure 7a). However, higher magnification indicates a great amount of smaller fractions, among them being gold clusters (Figure 6b,c) and ultra-small 1–2 nm nanoparticles (Figure 7b,c).

Additional tests were made using eugenol as a reducing agent of Au(III) ions. The UV-Vis spectrum was collected and shown in Supporting Materials (Figure S3). The eugenol solution was mixed with a solution of Au(III), and within 15 s the yellow color changed to colorless, suggesting a reduction in Au(III) to Au(I) ions. Then, within 6 min, a dark brown color in the solution was observed, confirming the appearance of the metallic phase of Au. Further changes in color were observed, leading to the formation of a black precipitate.

The second component of cinnamon extract is cinnamaldehyde. Lee et al. [35] developed a one-pot synthesis of AuNPs using cinnamaldehyde as a reducing agent. The reductant was dissolved in warm ethanol, whereas Au(III) ions were dissolved in water, then mixed and stirred at 100 °C. The red color of colloidal gold appeared after one minute. In order to confirm or reject the role of cinnamaldehyde as a reductant, the synthesis of gold nanoparticles was carried out. The UV-Vis spectra of cinnamaldehyde were given in Supplementary Materials (Figure S3). When mixing cinnamaldehyde and Au(III), the color of the solution remained unchanged. During next few days, there were still no changes registered. This fact allows us to exclude this component as a reductant during the reaction condition. However, it cannot be excluded that this compound might take part in the process of gold nanocluster stabilization. This result also explained why eugenol leads to a precipitate formation, in contrast to the synthesis carried out using cinnamon extract. 

### 2.7. The Possible Mechanism of Metastable Clusters, Cluster Aggregates and Ultra-Small Particles Formation—HRSTEM vs. Spectrophotometry Analysis

**Figure 9:**
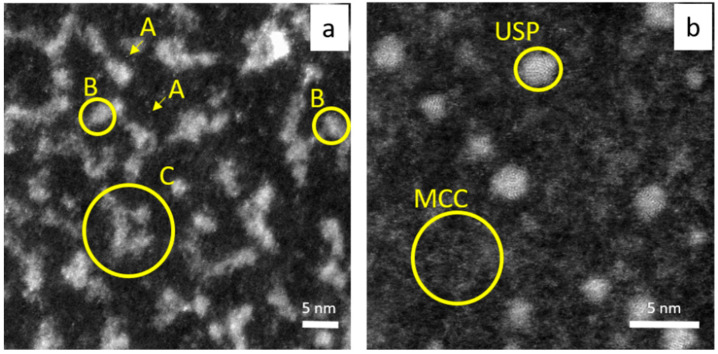
HRSTEM analysis of AuNC synthesized at 20 °C (**a**); 60 °C (**b**). Conditions: C_0, Au(III)_ = 0.01 M, C_0,Cex_ = 18 g/L. A—small Au cluster (few atoms), B—fragment of dense packed cluster (DPC), C—Au cluster aggregates; USP—ultra- small particles, MCC—metastable cluster clouds.

The results obtained from HRSTEM analysis give us a more complete overview about what happened in the colloidal system compared to the spectrophotometry study. Moreover, the progress in the process of nanoclusters, clusters aggregates, ultra-small and 10–20 nm gold nanoparticle formation and crystal growth have been captured. Surprisingly, at 20 °C, it was mainly aggregates of gold nanoclusters (dense clusters packing, DCP, see, Figure 9a) dispersed in low density clusters matrices (metastable cluster clouds, MCC) that were observed, whereas, at 60 °C, ultra-small gold particles (USP, see Figure 9b) surrounded by a MCC (see, Figure 9b) were visible. Considering this, that process of Au nanoparticle formation was carried out at the same reaction condition (composition), and we assumed that different temperatures allowed us to “catch” different steps in the process of particles evolution. The appearance of different forms of the same element in the analyzed sample (small Au cluster, cluster aggregates, ultra-small particles, Figure 9, large particles, Figure 6a and Figure 7a) suggests that the colloidal system is a dynamic system in which more than one stage may exist (being in equilibrium). 

The question is as follows: why do clusters aggregate (Figure 9a), and why are they observed only in the sample obtained at 20 °C, whereas USP (Figure 9b) are only in the sample obtained at 60 °C? These differences might suggest different growth mechanisms in the process of nanoparticle formation (D > 2 nm). At lower temperatures, the coalescence of the core in the DPC was observed, whereas, at 60 °C, Ostwald ripening played a main role in the process of USP and gold nanoparticle formation.

**Figure 10:**
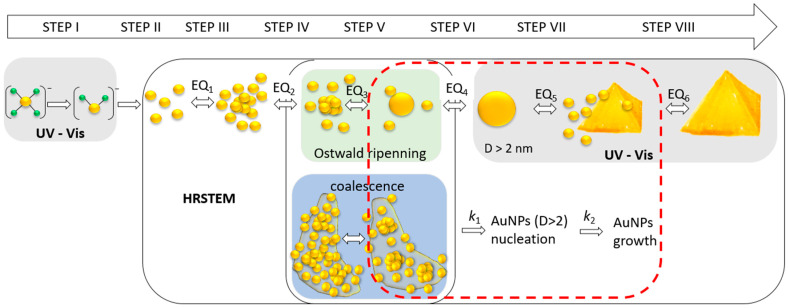
The proposed mechanism of Au nanoparticles formation based on HRSTEM results with the highlighted range of available techniques used in this work. Notation (from left): Au(III) ions (yellow—gold; green—chloride); Au(I) ions; free gold atoms; metastable clusters; dense packed cluster aggregates with core; small particles, D < 2 nm (only HRSTEM), nanoparticles with size D > 2 nm (UV-Vis and HRSTEM), crystal growth (D < 100 nm); crystal further growth, D > 100 nm (turbidity on UV-Vis spectrum). EQ_1–6_—dynamic equilibria. UV—wavelength below 400 nm, Vis—wavelength in the range 400–900 nm.

Based on the obtained results, the following mechanism scheme, shown in Figure 10, is proposed.

The scheme of this mechanism contains at least eight steps, which describe the processes taking place in the colloidal solution. They are the following:

Steps I, II: The reduction in Au(III) to Au(0) according to Equations (15)–(17);

Step III: Atoms association and metastable cluster formation;

(18) $$nAu\left(0\right)\overset{k_{C1}}{\to }{Au(0)}_{n}$$

Step IV: The autocatalytic clusters growth and formation of the core;

(19) $${Au(0)}_{n}+Au\left(0\right)\overset{k_{C2}}{\to }{Au(0)}_{n+1}$$

Step V: Ultra-small particles formation (1–2 nm, in diameter) or core coalescence;

(20) $${Au(0)}_{n+1}+{Au(0)}_{n}\overset{k_{C3}}{\to }USP$$

Step VI: Nanoparticles formation (D > 2 nm) via Ostwald ripening or coalescence;

(21) $$USP+Au\left(0\right)/{Au(0)}_{n}\overset{k_{P1}}{\to }AuNPs\;\mathrm{or}\;{nAu(0)}_{n+1}\overset{k_{P1}}{\to }\;AuNPs$$

Step VII: Particles autocatalytic growth and larger particle formation, having several possible shapes: spherical, rods, triangular, etc. (size: D < 100 nm)

(22) $$nAuNPs+Au\left(0\right)/{Au\left(0\right)}_{n}\overset{k_{P2}}{\to }{AuNPs}_{n}$$

where: *k_C_*_1_—kinetic rate constant of metastable cluster formation; *k_C_*_2_—kinetic rate constant of autocatalytic cluster growth, *k_C_*_3_—kinetic rate constant of USP nucleation; *k_P_*_1_—kinetic rate constant of USP growth and NPs formation (D > 2 nm); *k_P_*_2_—kinetic rate constant of autocatalytic growth of nanoparticles; *Au*(0)—gold atoms; *Au*(0)*_n_*—gold clusters; *Au*(0)*_n_*_+1_—gold clusters with the core; *USP*—ultra-small particles; *AuNPs*—gold nanoparticles; *AuNPs_n_*—larger gold nanoparticles.

The first two steps were described in Section 2.6. The third stage relates to atoms’ association due to Van der Waals forces [36,37] and the process of metastable cluster formation. These clusters can break or further grow and form densely packed aggregate clusters with the core (step IV). It seems also that clusters have a thin structure which has a wafer-like structure (1D) (see, Figure S5, Supplementary Materials). They can assume a size of approx. 10 nm and an irregular shape (which, again, suggests an association of smaller gold clusters). Only the appearance of the core causes the structure to grow in the 2D direction, leading to USP formation (see, Figure 8B) [38] via the Ostwald ripening mechanism. This process was observed using HRSTEM at 60 °C, whereas, at 20 °C, the coalescence of clusters cores was observed. Both mechanisms lead to nanoparticle formation (D > 2 nm) (step VI).

In the next step, the substrates for the growth of nanoparticles are metastable clusters that form (MCC) around the particles (see, Figure S6a,b, Supplementary Materials) and aggregates which can also diffuse to the surface of nanoparticles and build into their structure (see, Figure S6c,d, Supplementary Materials).

The steps (III–VIII) can be observed using the HRSTEM technique, whereas spectrophotometry in the visible wavelength range can be applied for nanoparticles (>2 nm) that exhibit an LSPR effect. The larger crystals (>100 nm, step VIII, Figure 10) causes turbidity, which results in the increase in the spectrum over the entire wavelength range.

### 2.8. The Impact of the Beam Irradiation on Gold Cluster Transformation—Single and Multi-Core Formation

The influence of the beam irradiation on metal cluster transformation during HRSTEM analysis was studied by Henninen et al. [38]. They showed in situ platinum cluster evolution leading to the cluster coalescence and particle formation. In our studies, we observed an internal gold core formation at the expense of cluster breakup (see, Supplementary Movie). Another interesting observation was related to the core formation in cluster aggregates. Figure S7 (Supporting Materials) indicates a multi-core formation within nanocluster aggregates. Consequently, irradiation leads to the nearest core’s coalescence and the formation of polycrystals. It suggests that the presence of multicores is responsible for polydispersity in the formed particles.

## 3. Materials and Methods

### 3.1. Chemicals

As a metal precursor, a base solution 0.1 M HAuCl_4_ (pH = 1, 0.1 M HCl as a solvent (preventing gold hydrolysis) was used. Cinnamon extract was used as an Au(III) ion bio reductant and particle stabilizer. For this purpose, 2 g of cinnamon powder (bark powder commercially available in the market, Prymat, Koszalin, Poland) was dispersed in 100 mL of deionized water (concentration—20 g/L). The solution was mixed (360 rpm, T = 20 °C) for 24 h according to procedure described by Siemieniec and Kruk [21]. Investigations were performed at a constant temperature in the range of 20–60 °C. Afterward, the solution underwent a two-stage filtration process with middle pore sized paper and a syringe filter (0.2 µm). After this process, the solution had an intense yellow color and was ready to use.

Preparation of the base solution of Eugenol (Eug). For this purpose, the 10 µL of eugenol (99%, p.a., Thermo Scientific, Waltham, MA, USA) was dissolved in 10 mL of deionized water and was treated as the base solution of the reductant. Taking into account that eugenol is slightly soluble in water (2.46 g/L, d = 1.067 g/mL), we calculated the amount of proper volume, and it is equal to 2.3 mL which is able to dissolve in 1 L. For preparation of the base solution of eugenol, a lower volume was taken, i.e., 10 µL (99%) which was next dissolved in 10 mL of water. The concentration of Eug equals c.a. 6 mM.

Preparation of base solution of Cinnamaldehyde (CA). For this purpose, the 10 µL (98%, p.a.) was dissolved in 10 mL of deionized water and was treated as a base solution of the reductant. The value 10 µL was calculated based on information about the solubility in water (1.084 g/L, d = 1.041 g/mL, T = 20 °C). The initial concentration of CA equals c.a. 8 mM.

### 3.2. Gold Nanoparticles Synthesis

Gold nanoparticle synthesis was carried out in the batch reactor. For this purpose, 1 mL of 0.1 M gold solution was added to 9 mL 20 g/L fresh filtered cinnamon extract and mixed using a magnetic stirrer (360 rpm). Depending on the temperature, the solution color change was observed after a different period of time, suggesting the process of nanoparticle formation.

### 3.3. Methods of Analysis

The process of gold nanoparticle formation was investigated using UV–Vis spectrophotometer UV-2501PC (Shimadzu, Kyoto, Japan), working in the wavelength range of 190–900 nm and equipped with thermostated cell. Spectrophotometer was equipped in Double monochromator with a high-performance double-blazed holographic grating in the aberration corrected Czerny-Turner mounting. This spectrophotometer enabled the recording of an absorbance of up to 4 a.u. with a small error (1%), whereas, at value 5 a.u., this error equals 6%. Thus, during experiments, the spectra intensity registered up to 4 a.u. Then, in order to collect a more intense spectra, we exchanged the path length of the cuvette from L = 10 mm on L = 2 mm, while, for L = 2 mm, we obtained Amax ≈ 1 a.u. In order to compare the spectrum obtained in this way with the remaining results, we multiplied the absorbance value by five. In the typical procedure, after reagent mixing, i.e., Au(III) ions and cinnamon extract (see, Section 2.2), the 3 mL of the colloidal solution was immediately introduced into a quartz cuvette (Hellma, optical path = 1 cm or 2 mm) and thermostatic cell. The spectra were recorded using UV Probe Software, whereas results analyses were performed using Origin Pro 8.5.

An advanced, probe Cs-corrected scanning transmission electron microscope (Titan3 G2 60–300, Thermo Fisher Scientific, Bleiswijk, The Netherlands) was used to image the morphology of the material to collect the data for the size distribution measurements and to confirm the crystallographic structure of the obtained particles. The latter one was supported by the JEMS software (https://www.jems-swiss.ch/ (accessed on 29 February 2024)). For the HRSTEM analysis, 100 µL of colloidal gold was dropped at a copper grid covered with carbon film.

A fluorescence study was carried out using spectrofluorometer FS5 (Edinburgh Instruments, Livingston, UK). The excitation wavelength was set at 320, 350 and 400 nm, and emission was observed in the range of 400–540 nm, with maximum intensity at 455 nm.

## 4. Conclusions

The process of AuNPs formation was carried out using green reductants, such as cinnamon extract containing eugenol responsible for Au(III) ion reduction, with cinnamaldehyde as a possible stabilizer. The nucleation and growth rate constants of nanoparticles (D > 2 nm) were determined at different temperatures using the Watzky–Finke model. The values of energy, enthalpy, and entropy of activation for each stage related to the process of nanoparticle formation (index 1 relates to nucleation and 2 to the growth): E_1_ = 70.6 kJ, E_2_ = 19.6 kJ, ΔH_1_ = 67.9 kJ/mol, ΔH_2_ = 17 kJ/mol, ΔS_1_ = −76.2 J/(K·mol), ΔS_2_ = −204.2 J/(K·mol) were determined. A HRSTEM analysis showed that, depending on the temperature of the synthesis process, the transformation of the Au cluster into nanoparticles can be observed. Densely packed clusters with core undergoes coalescence, which leads to formation of nanoparticles with size D > 2 nm. At 60 °C, particles that were ultra-small in size, i.e., 1–2 nm (in diameter), and spherical in shape were observed (Figure 7c). Then, the USP growth occurred because of Ostwald ripening.

Moreover, the obtained USP emits light at a wavelength of 455 nm during excitation at 320 nm, whereas at lower temperatures, i.e., 20 °C the formation of 10–20 nm (diameter), irregular in shape gold clusters were determined (Figure 6c). The selection of techniques (UV-Vis spectroscopy, HRSTEM) and experimental conditions (composition, temperature) allowed us to screen the colloidal system and take a look at the mechanism of nanoparticle formation, showing the possible coexistence of intermediates which are in dynamic equilibrium. Based on HRSTEM and spectrophotometric studies, we propose a more complex (at least 8—steps, Figure 10) path in the process of gold nanoparticle formation while considering two mechanisms leading to particle growth: coalescence and Ostwald ripening.

## Figures and Tables

**Table 1:** Most important parameters, i.e., *t*_max_, ${\left[B\right]}_{t_{\max}}$, *t_in_*_(*jerk*)_ and formulas needed for kinetics rate constants of nucleation and particles growth determination.

Parameter	Formula	Equation No.
*t*_max_	$t_{\max}=\frac{\ln(\frac{k_{2}{\left[A\right]}_{0}}{k_{1}})}{k_{2}{\left[A\right]}_{0}}$	(10)
${\left[B\right]}_{t_{\max}}$	${\left[B\right]}_{t_{\max}}=\frac{{\left[A\right]}_{0}}{2}$	(11)
*t_in_*_(*jerk*)_	$t_{in(jerk)}=t_{\max}-\frac{1.3}{k_{2}{\left[A\right]}_{0}}$	(12)

**Table 2:** The values of obtained rate constants for slow continuous nucleation and fast autocatalytic growth *. Conditions: C_0, Au(III)_ = 0.01 M, C_0,Cex_ = 18 g/L.

T, °C	*t*_max_, min	*t_in_*_(*jerk*)_, min	*k*_1_·10^3^, min^−1^	*k*_2_·10^2^, (a.u.)^−1^min^−1^
20	12	9.87	0.4	12.11
30	8	6.40	1.2	16.11
40	6	4.44	5.6	16.53
50	4	3.00	7.2	25.79
60	3	2.20	12.4	32.24

* For ${\left[B\right]}_{t_{\max}}$ = 2.52 (a.u.), the values of [*A*]_0_ = 5.04 (a.u).

**Table 3:** The values of obtained rate constants for slow continuous nucleation and fast autocatalytic growth. Conditions: C_0, Au(III)_ = 0.01 M, C_0,Cex_ = 18 g/L, T = 20–60 °C.

Process	*A*, dm^3^mol^−1^min^−1^	*E_a_*, kJ	∆S, JK^−1^mol^−1^	∆H, kJ mol^−1^
nucleation	1.3 × 10^9^	70.6	−76.2	67.9
growth	4.0 × 10^2^	19.6	−204.2	17.0

## Data Availability

Data available on request.

## Supplementary Materials

The following supporting information can be downloaded at: https://www.mdpi.com/article/10.3390/molecules29071426/s1, S1—Topic analysis. S2—Deconvolution of the cinnamon extract, spectrum of cinnamaldehyde and eugenol spectrum. S3—The determination of the characteristic time: t_max_, t_in_ and t_in(jerk)_. S4—HRSTEM analysis. S5. HRSTEM movie animation description and movie. Figures: Figure S1: Statistic data: the numbers of papers (number of publications by topic: Fluorescence; Metal Clusters; Icosahedron) published within 10 years (a), Publication share by subject area (b). Source: Scopus database (SciVial); Figure S2: The deconvolution of the cinnamon extract spectrum, 100,000 dissolutions of base solution in H_2_O, T = 20 °C; Figure S3: The spectrum of cinnamaldehyde (CA) and eugenol (Eug) after 100,000 dissolutions of base solutions (CA, >98%, Fluka; Eug, 99%, p.a., Thermo Scientific) in H_2_O; Figure S4: The first (a), second (b) and third-order (c) derivative of B in time function. The kinetic curve registered at 538 nm (d) with highlighted points on the time scale. Conditions: C_0, Au(III)_ = 0.01 M, C_0,Cex_ = 18 g/L, T = 40 °C; Figure S5: The HRSTEM analysis showing a gold cluster structure. Conditions: C_0, Au(III)_ = 0.01 M, C_0,Cex_ = 18 g/L, T = 20 °C; Figure S6: The HRSTEM analysis showing a fragment of the larger gold crystal with a marked l5 nm thick layer (A) of gold nanoclusters aggregates and/or MCC (a); the fragment of the larger gold crystal with marked (B) ultra-small gold particles (b); gold nanoparticles with irregular shapes (c) and exemplary particles with a gray border around the particle (black circles); magnification of cluster aggregates (d). Conditions: C_0, Au(III)_ = 0.01 M, C_0,Cex_ = 18 g/L, T = 60 °C (a,b). T = 20 °C (c,d); Figure S7: The HRSTEM analysis showing the coalescence of cores within gold nanoclusters aggregates at a beginning (a) and after irradiation (b). A–A’—coalescence of two cores, B–B’—coalescence gold clusters and/or aggregated gold clusters leading to a new core formation. Conditions: C_0, Au(III)_ = 0.01 M, C_0,Cex_ = 18 g/L, T = 20 °C (a,b). Figure S8: Fluorescence spectrum obtained at different excitation wavelengths. Sample notation: A_0—colloidal solution after one year; A_1—colloidal solution after 10-times dissolution. Conditions: C_0, Au(III)_ = 0.01 M, C_0,Cex_ = 18 g/L, T = 60 °C. Figure S9: A: HRSTEM image with selected analyzed area, B: FFT analysis with selected spots (1-1, 2-2, 3-3), C1–C2: Mask used to select certain spots for IFFT, D1–D2: IFFT image using indices chosen by C1–C2 mask, E1–E2: lattice parameter measurement through measuring the distance of white fringes on IFFT (D1–D2) images. Conditions: C_0, Au(III)_ = 0.01 M, C_0,Cex_ = 18 g/L, T = 20 °C. Reference [39] is cited in the Supplementary Materials.
