# Supplementary material for: Synthesis of Gold Clusters and Nanoparticles Using Cinnamon Extract—A Mechanism and Kinetics Study
Source: Molecules. 2024 Mar 22;29(7):1426. doi: 10.3390/molecules29071426 (PMC11013221; doi:10.3390/molecules29071426)
Supplement: Supplementary file 1 [file molecules-29-01426-s001.zip › Supplementary Materials.pdf]

Supporting Materials

Synthesis of Gold Clusters and Nanoparticles Using Cinnamon Extract—A Mechanism and Kinetics Study

Magdalena Luty-Błocho [1,*], Jowita Cyndrowska [1], Bogdan Rutkowski [2] and Volker Hessel [3]

[1] AGH University of Krakow, Faculty of Non-Ferrous Metals, al. A. Mickiewicza 30, 30-059 Krakow, Poland; cyndrowska@student.agh.edu.pl
[2] AGH University of Krakow, Faculty of Metals Engineering and Industrial Computer Science, al. A. Mickiewicza 30, 30-059 Krakow, Poland; rutkowsk@agh.edu.pl
[3] School of Chemical Engineering, The University of Adelaide, Adelaide, SA 5005, Australia; volker.hessel@adelaide.edu.au
* Correspondence: mlb@agh.edu.pl

S1. Topic analysis

Recently, a new trend (see, Fig. S1a) related to the synthesis and application of gold nanoclusters (AuNCs) in different areas appeared, especially in chemistry, materials science, chemical engineering, medicine [39], etc. (see, Fig. S1b). The statistical data analysed based on Scopus database (SciVial, 14th December) shows that searching by Topic T.1445: Fluorescence; Metals Clusters; Icosahedron being a part of the Topic Cluster TC.47 – Plasmonic; Materials; Surface Plasmon Resonance, is located on 99.804 Topic Prominence percentile.

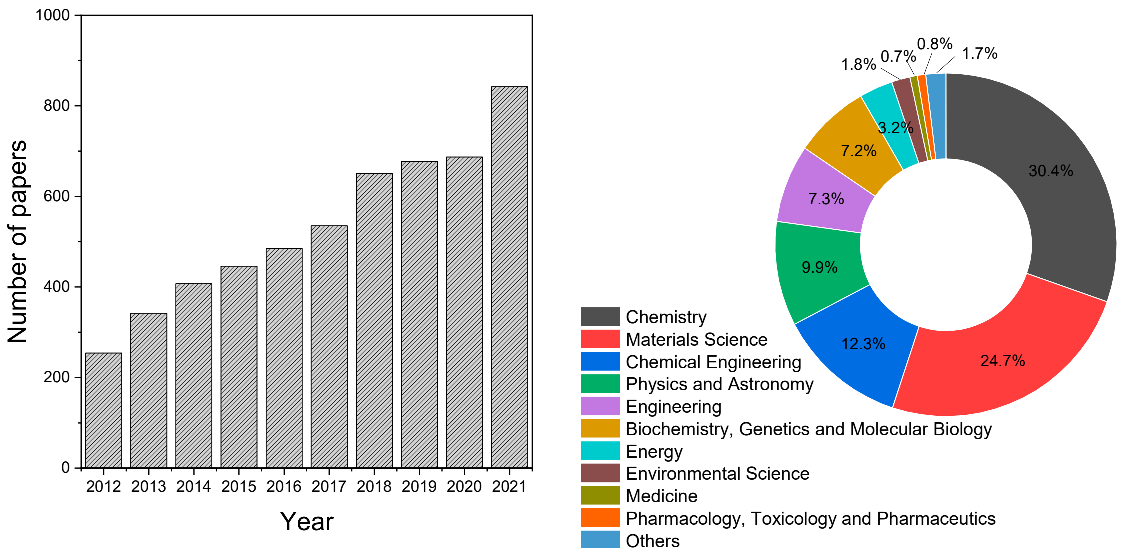

(a) (b)

Figure S1. Statistic data: the numbers of papers (number of publications by Topic: Fluorescence; Metal Clusters; Icosahedron) published within 10 years (a), Publication share by subject area (b). Source: Scopus database (SciVial).

Moreover, the analysis of the top keyphrase by relevance, based on 5 325 publication (2012-2021) shows that "nanoclusters" are on the top, whereas "gold nanoclusters" are on the second place, confirming the importance of this topic.

S2. Deconvolution of the cinnamon extract, spectrum of cinnamaldehyde and eugenol spectrum.

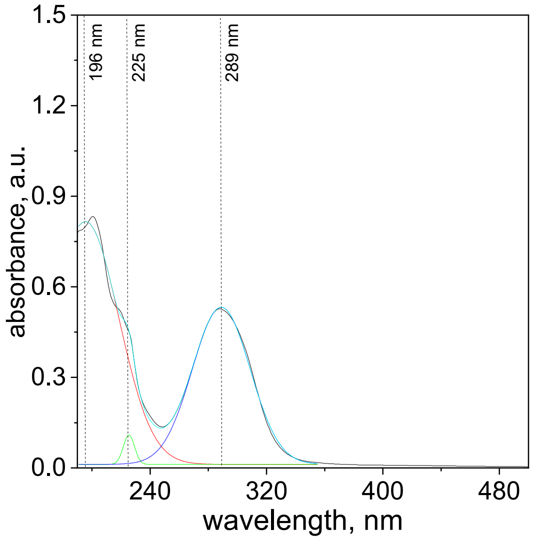

Figure S2. The deconvolution of the cinnamon extract spectrum, 100 000 dissolutions of base solution in H_2O, T = 20°C. Black line – experimental spectrum, blue line – cumulative feat peak, green line – peak at 225 nm, dark blue line – peak at 289 nm, red line – peak at 196 nm.

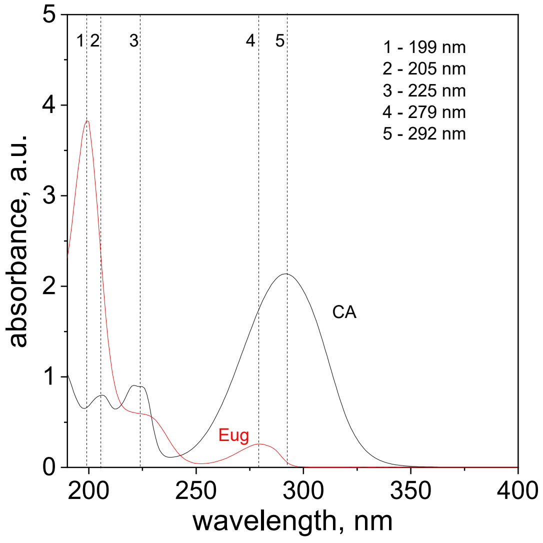

Figure S3. The spectrum of cinnamaldehyde (CA) and eugenol (Eug) after 100 000 dissolutions of base solutions (CA, >98%, Fluka; Eug, 99%, p.a., Thermo Scientific) in H_2O.

S3. The determination of the characteristic time: t_{max}, t_{in} and $t_{in(jerk)}$.

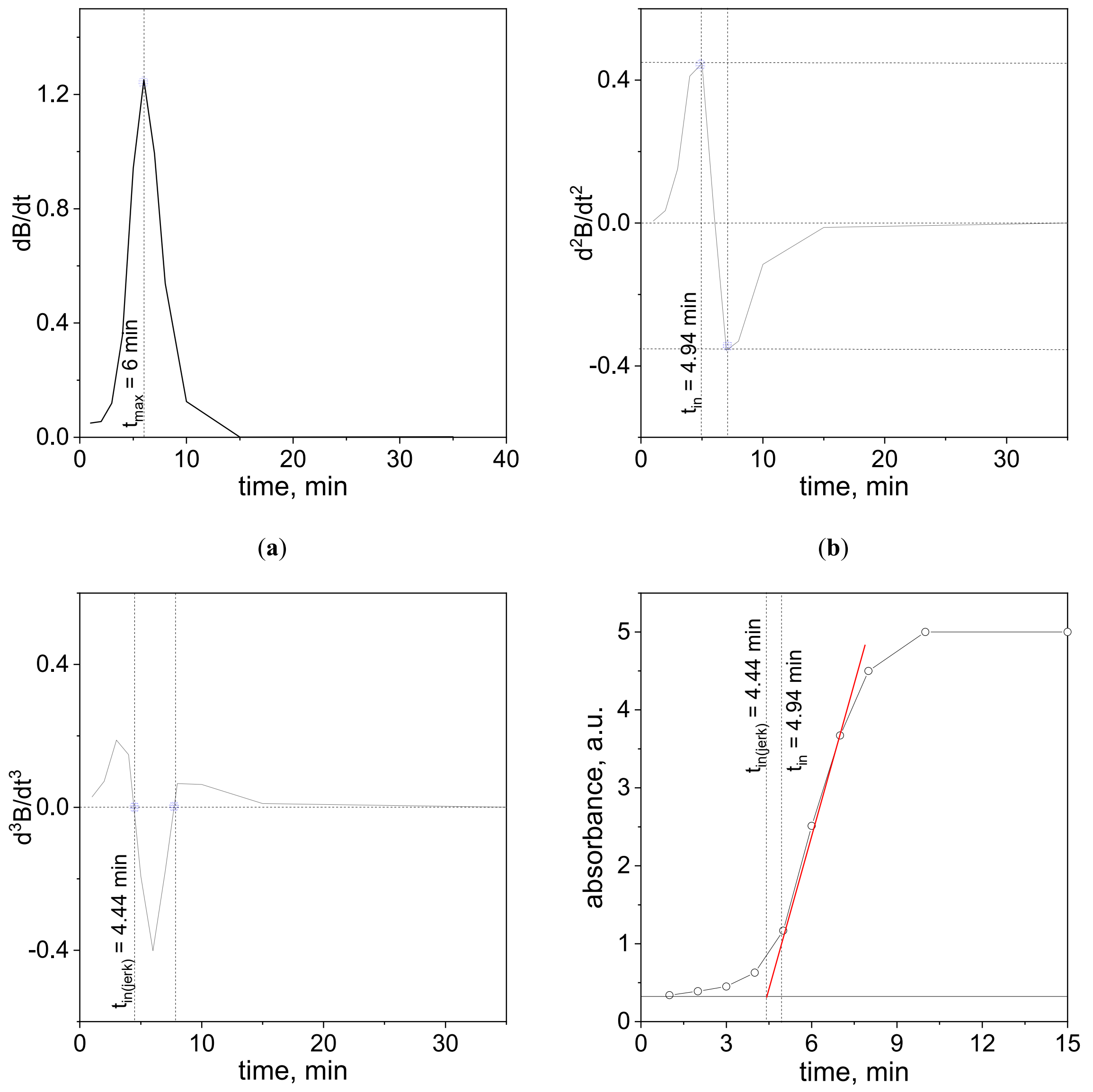

(c) (d)

Figure S4. The first (a), second (b) and third-order (c) derivative of B in time function. The kinetic curve registered at 538 nm (d) with highlighted points on the time scale. Conditions: $C_{0,\ Au(III)} = 0.01$ M, $C_{0,Cex} = 18$ g/L , T = 40°C.

S4. HRSTEM analysis

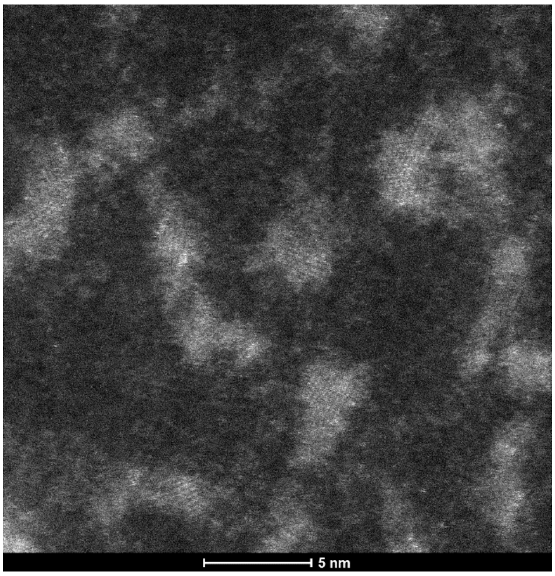

Figure S5. The HRSTEM analysis showing gold cluster structure. Conditions: $C_{0,\ Au(III)}$ = 0.01 M, $C_{0,Cex}$ = 18 g/L , T = 20°C.

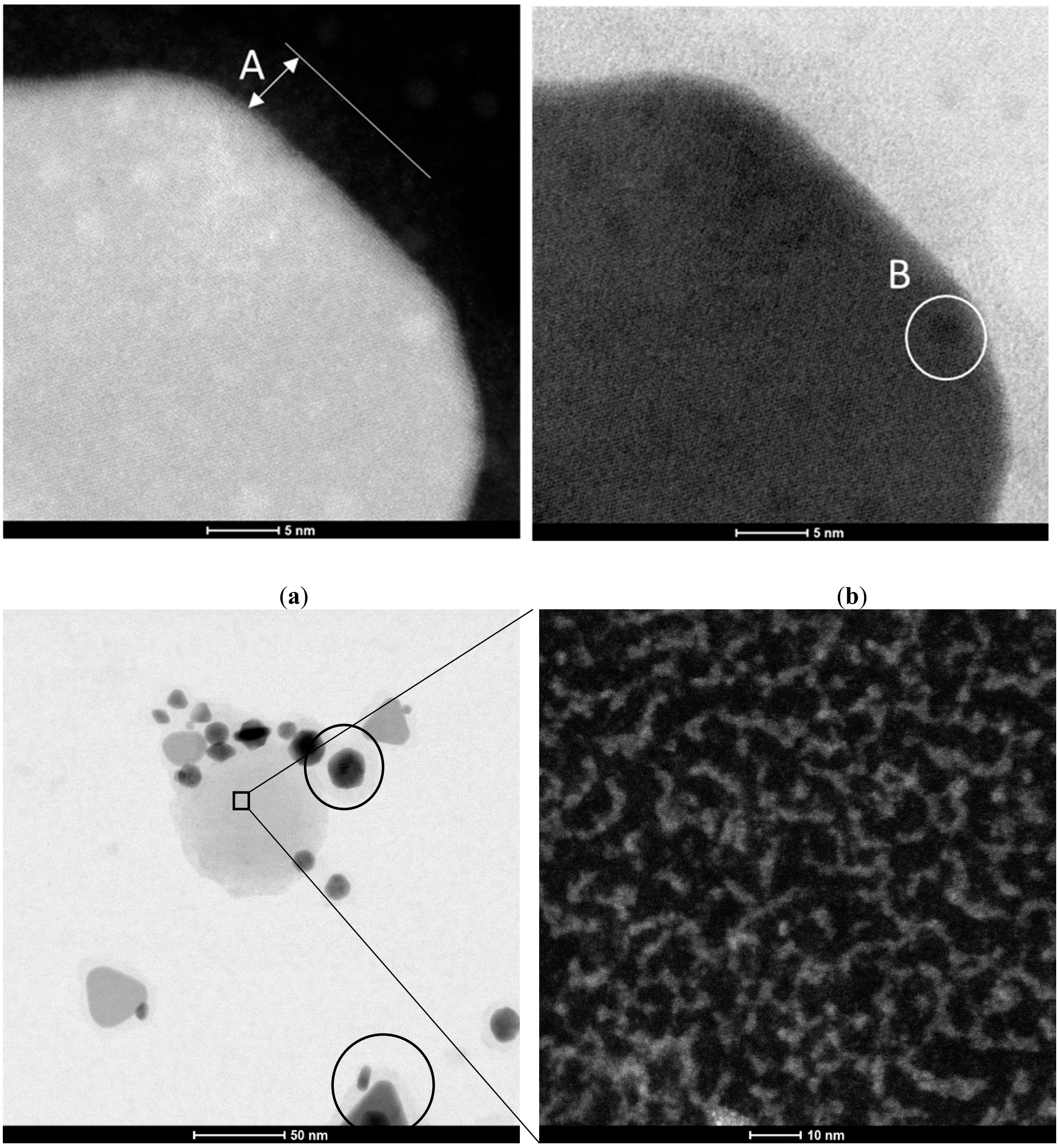

(c) (d)

Figure S6. The HRSTEM analysis showing a fragment of the larger gold crystal with marked 15 nm thick layer (A) of gold nanoclusters aggregates and/or MCC (a); the fragment of the larger gold crystal with marked (B) ultra-small gold particles (b); gold nanoparticles with irregular shapes (c) and exemplary particles with a gray border around the particle (black circles); magnification of cluster aggregates (d). Conditions: $C_{0,\ Au(III)}$ = 0.01 M, $C_{0,Cex}$ = 18 g/L , T = 60°C (a, b). T = 20°C (c, d).

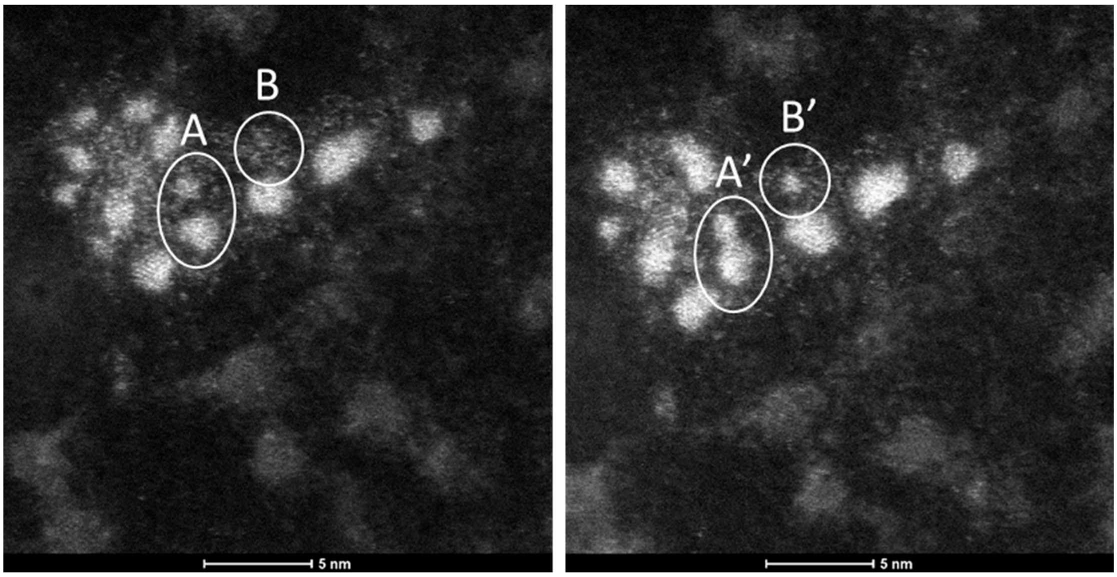

Figure S7. The HRSTEM analysis showing coalescence of cores within gold nanoclusters aggregates at a beginning (a) and after irradiation (b). A – A' – coalescence of two cores, B – B' – coalescence gold clusters and/or aggregated gold clusters leading to a new core formation. Conditions: $C_{0,\ Au(III)}$ = 0.01 M, $C_{0,Cex}$ = 18 g/L , T = 20°C (a, b).

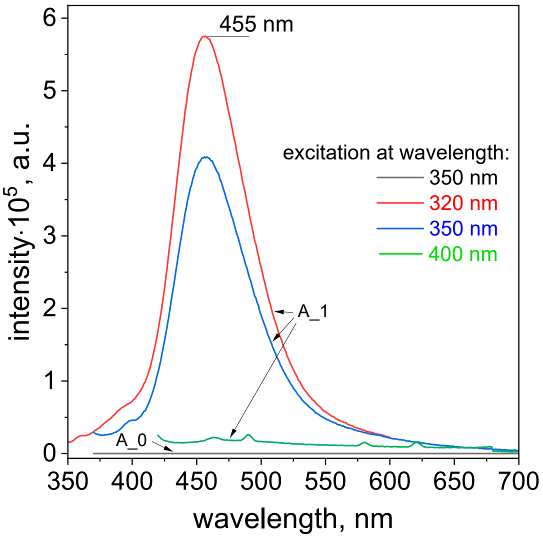

Figure S8. Fluorescence spectrum obtained at different excitation wavelengths. Sample notation: A_0 – colloidal solution after one year; A_1 – colloidal solution after 10-times dissolution. Conditions: $C_{0,\ Au(III)}$ = 0.01 M, $C_{0,Cex}$ = 18 g/L , T = 60°C.

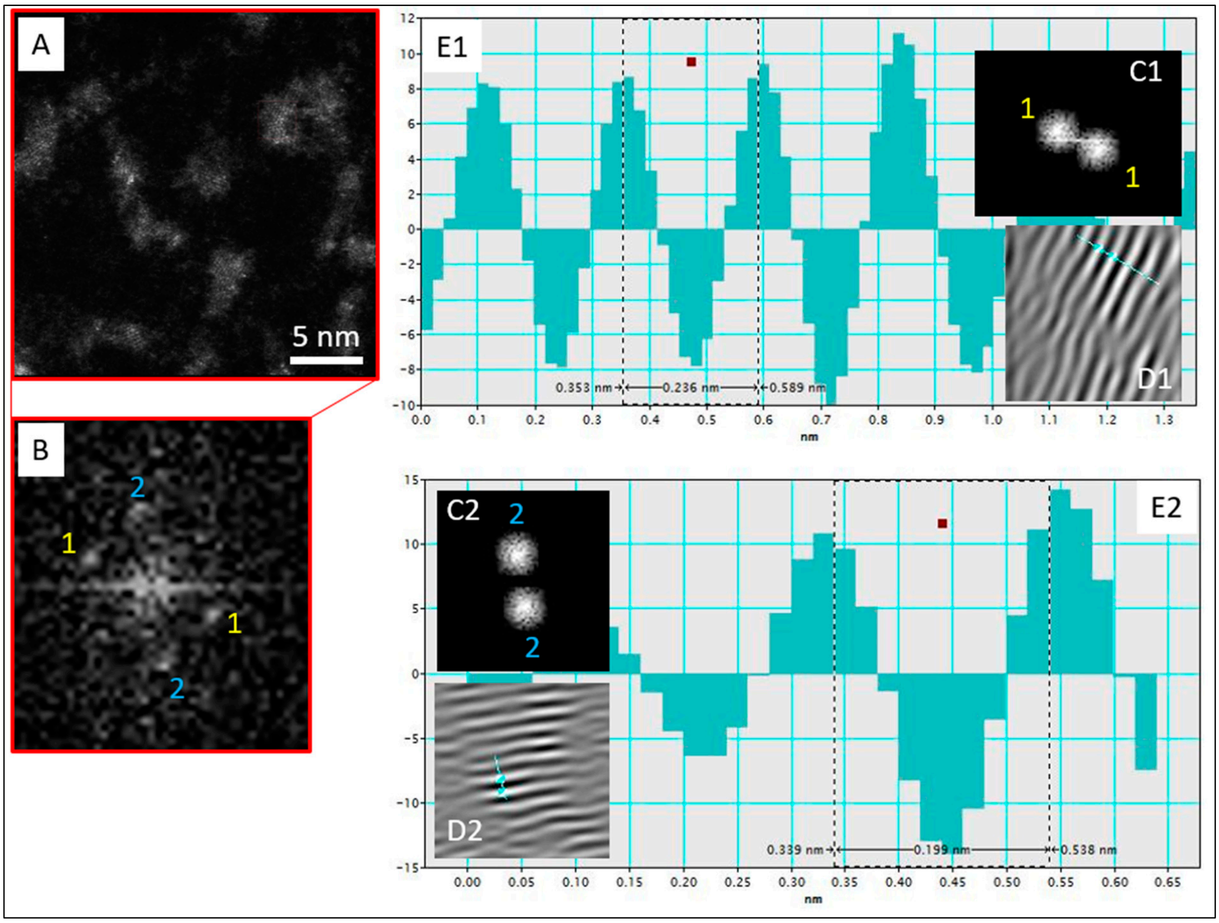

Figure S9. A: HRSTEM image with selected analyzed area, B: FFT analysis with selected spots (1-1, 2-2, 3-3), C1-C2: Mask used to select certain spots for IFFT, D1-D2: IFFT image using indices chosen by C1-C2 mask, E1-E2: lattice parameter measurement through measuring the distance of white fringes on IFFT (D1-D2) images. Conditions: $C_{0,\ Au(III)}$ = 0.01 M, $C_{0,Cex}$ = 18 g/L, T = 20°C.

S5. HRSTEM movie animation

The movie demonstrating multi-core coalesce during HRSTEM analysis (under beam irradiation) was constructed based on 50 photos using Google photos application.

Reference

[39] van de Looij, S.M.; Hebels, E.R.; Viola, M.; Hembury, M.; Oliveira, S.; Vermonden, T. Gold Nanoclusters: Imaging, Therapy, and Theranostic Roles in Biomedical Applications. *Bioconjugate Chem.* **2022**, *33*, 4–23. https://doi.org/10.1021/acs.bioconjchem.1c00475.
